# The molecular landscape of nodular fasciitis: novel USP6 fusions, genomic stability, and an exploratory clinicopathological model – a 20‐year retrospective study

**DOI:** 10.1002/2056-4538.70117

**Published:** 2026-10-09

**Authors:** Kristijan Skok, Maximilian T Buchwald, Maria Anna Smolle, Theresa Godschachner, Andreas Leithner, Christian Viertler, Bernadette Liegl‐Atzwanger

**Affiliations:** ^1^ Diagnostic and Research Institute of Pathology Medical University of Graz Graz Austria; ^2^ Department of Orthopaedics and Trauma Medical University of Graz Graz Austria

**Keywords:** nodular fasciitis, *USP6* gene fusion, soft tissue neoplasms, molecular diagnostics, histopathology, aggressive behavior, novel gene fusions, rare partners, diagnostic pitfalls

## Abstract

Nodular fasciitis (NF) is a benign myofibroblastic neoplasm characterized by rapid growth and recurrent *USP6* rearrangements. Despite its benign nature, NF may exhibit worrisome histologic or clinical features that mimic sarcoma, posing diagnostic challenges. The relationship between molecular diversity and morphologic heterogeneity remains incompletely understood. We retrospectively analyzed 283 NF cases diagnosed between 2000 and 2024 at a tertiary referral center. Molecular testing for *USP6* rearrangements was performed in 44 cases using targeted RNA sequencing. Histologic features, clinical parameters, and fusion partner distribution were evaluated, with emphasis on lesions showing infiltrative or worrisome morphology. Selected cases underwent additional genomic profiling, including hybrid‐capture DNA sequencing and copy‐number analysis. Among 44 molecularly confirmed cases, 18 distinct *USP6* fusion partners were identified. The most frequent fusion was *MYH9*::*USP6* (40.9%), followed by *COL1A1*::*USP6*, *MIR22HG*::*USP6*, and *RRBP1*::*USP6*. Rare and previously unreported fusions, including *CLK1*::*USP6* and *FOSL2*::*USP6*, further expanded the molecular spectrum. Two cases with infiltrative, aggressive‐appearing morphology showed no additional pathogenic alterations and a genomically stable profile. Based on these findings, we propose an exploratory, hypothesis‐generating framework to summarize the observed clinicopathological spectrum of NF. NF demonstrates substantial molecular heterogeneity but retains a genetically simple and largely benign biological behavior. Even lesions with alarming morphology appear genomically stable and clinically indolent. The proposed framework may facilitate diagnostic interpretation and provide a conceptual framework for future studies but requires validation before any biological or clinical implications can be inferred.

## Introduction

Nodular fasciitis (NF) represents a benign mesenchymal neoplasm that typically arises within the superficial soft tissues of young adults [1]. NF was at first described as a pseudosarcomatous lesion in the 1960s [2]. Due to its rapid growth, NF may clinically resemble a malignant neoplasm; however, it is a benign lesion that may regress spontaneously [3, 4]. The upper extremities, the head and neck region, and the trunk constitute the most common localizations, although virtually any anatomical site can be affected [3, 5, 6]. Moreover, histologically it can imitate a malignant process due to high cellularity, frequent mitotic figures, and a focal infiltrative pattern. Diagnostic challenges occur mainly in the evaluation of infiltrative lesions or in small tissue biopsies [7]. Molecular investigations over the past decade have revealed that the great majority of NF lesions, estimated at more than 90%, harbor a rearrangement of the *USP6* (ubiquitin‐specific protease 6) gene, resulting in overexpression of the encoded deubiquitinating enzyme [3, 8]. *USP6* rearrangements have also been identified in several other benign mesenchymal lesions, collectively referred to as *USP6*‐associated neoplasms, including aneurysmal bone cyst (ABC) [9], myositis ossificans (MO), fibro‐osseous pseudotumor (FOP) of the digits, cranial fasciitis, and fibroma of the tendon sheath [10, 11, 12, 13]. They typically harbor characteristic gene fusions in which the *USP6* coding region is placed under the control of various partner gene promoters, resulting in transcriptional upregulation of *USP6* [12]. Identification of *USP6* rearrangement has become an important diagnostic adjunct, especially in small tissue samples where morphology alone may be inconclusive, thereby helping to prevent unnecessary aggressive treatment [7]. Several *USP6* fusion partners have been reported, with *MYH9* being the most frequent. Additional partners include *RRBP1*, *MIR22HG*, *COL1A1*, *COL1A2*, *COL3A1*, *COL6A2*, *PAFAH1B1*, *CALD1*, *CALU*, *EIF5A*, *EMP1*, *NR1D1*, *SPARC*, *SERPINH1*, *THBS1*, *THBS2*, *SREBF1*, *PPP6R3*, *STAG1*, among others [14]. To date, more than 25 fusion partners have been identified, all resulting in transcriptional upregulation of *USP6* through promoter swapping [7].

This retrospective single‐center study was undertaken to characterize the clinicopathological and molecular spectrum of NF, with particular emphasis on lesions demonstrating pronounced infiltrative growth. The study aimed to investigate whether these uncommon infiltrative presentations were associated with additional molecular alterations. Considering prior reports of malignant behavior in morphologically classic lesions, we also systematically reviewed our archival material to identify comparable cases. Based on our clinicopathological and molecular observations, we propose a conceptual, hypothesis‐generating framework summarizing the observed clinicopathological spectrum.

## Materials and methods

### Case selection

A comprehensive search of our institutional database was performed to identify both in‐house and consultation cases with a confirmed diagnosis of NF from 2000 to 2024. In total, 283 NF cases were retrieved. In 44 cases molecular analysis was performed and demonstrated a *USP6* gene rearrangement. Molecular testing was not performed consecutively in all NF cases but was requested as part of routine diagnostic work‐up in cases where the diagnosis remained uncertain after conventional histopathological and immunohistochemical evaluation. Indications for RNA sequencing included unusual histomorphology, infiltrative growth, atypical anatomical location, limited biopsy material, discordance between morphology and clinical or radiological findings, or a differential diagnosis including malignant spindle‐cell neoplasms. To provide molecular context for the spectrum of *USP6*‐associated neoplasms, we additionally reviewed 12 non‐NF *USP6*‐rearranged lesions, comprising ABC (*n =* 8), MO (*n* = 2), fasciitis ossificans (FO; *n* = 1), and one lesion morphologically classified proliferative myositis‐like (PM; *n* = 1) identified in our institutional archives. These cases were analyzed descriptively and were not included in the primary analyses of NF. As the pathology institute functions as the national reference center for sarcoma pathology in Austria, nearly half of the cases (48.4%; 137/283) were consultation specimens referred from external institutions, highlighting the considerable diagnostic challenges associated with these tumors, particularly when evaluated on biopsy material (EK 1042/2025 and 32‐222 ex 19/20) (supplementary material, Table S1). Of the 44 molecularly investigated cases, 31 (70.5%) were consultation cases from external institutions, 6 (13.6%) originated from regional cooperation partners, and 7 (15.9%) represented cases from the Medical University of Graz (University Hospital).

Not all infiltrative lesions underwent molecular testing, particularly among older archival cases diagnosed before the routine implementation of RNA sequencing. However, as none of these cases showed recurrence, further retrospective molecular testing was not considered informative. The infiltrative cases included in the present study therefore reflect those for which molecular analysis was clinically indicated.

### Clinical and pathological data collection

Clinical, histopathological, and surgical data were retrieved from institutional archives and consultation reports. Extracted variables included age, sex, anatomical site, lesion size, surgical approach, and recurrence status. Histopathological parameters comprised matrix composition, growth pattern, cellular morphology, mitotic activity, and the presence of extravasated erythrocytes. Cases without molecular testing contributed to the description of the overall retrospective NF cohort. All tissue samples were formalin‐fixed paraffin‐embedded (FFPE). Diagnoses were established on hematoxylin–eosin (H&E) stained sections and supported by immunohistochemical profiling [strong expression of smooth muscle actin (SMA)] and molecular analysis.

### Histological review

Histologic material was obtained either through surgical excision or biopsy. All available slides were independently reviewed by at least three participating pathologists (BL‐A, CV, TG, and KS). Discrepant interpretations were resolved by joint review and consensus. The review included assessment of surgical margins, growth pattern, infiltrative growth pattern, cellular morphology, matrix quality, presence of extravasated erythrocytes, lesion size, and mitotic figures. In accordance with the histopathological features observed throughout the cohort, the reviewing pathologists agreed on the following operational definitions, which were subsequently used for the exploratory four‐class framework:

- Infiltrative growth was defined as irregular extension of lesional spindle cells into adjacent adipose tissue, skeletal muscle, fascia, or other native structures beyond the main tumor border.
- Aggressive morphology refers predominantly to architectural features suggestive of locally aggressive growth, including infiltrative margins, multinodularity, long sweeping fascicles, increased cellularity, and/or increased mitotic activity, while lacking unequivocal malignant cytological features.
- Sarcoma‐like morphology primarily refers to pronounced cytological atypia, including pleomorphism, marked nuclear atypia, and atypical mitotic figures, often accompanied by infiltrative growth and architectural features suggestive of malignancy.

### Molecular analysis

*USP6* rearrangements were investigated by targeted RNA sequencing. RNA was extracted using the Maxwell RSC RNA FFPE Kit on a Maxwell RSC 48 Instrument (Promega, Madison, WI, USA) and quantified using a NanoDrop ND‐1000 spectrophotometer (Thermo Fisher Scientific, Waltham, MA, USA). Anchored multiplex PCR‐based libraries were prepared initially using the in‐house‐modified Archer FusionPlex MUG Sarcoma Panel v4 (Diagnostic and Research Institute of Pathology, Medical University of Graz, Graz, Austria) and subsequently using the FusionPlex Sarcoma v2 or Pan Solid Tumor v2 panels for Ion Torrent (Integrated DNA Technologies, Coralville, IA, USA). Following quantification with the Ion Library TaqMan Quantitation Kit (Thermo Fisher Scientific), template preparation and chip loading were performed on the Ion Chef System, followed by sequencing on an Ion S5 XL or Ion GeneStudio S5 Prime instrument (Thermo Fisher Scientific).

Selected infiltrative cases underwent additional DNA profiling following extraction using established protocols [15]. Libraries were enriched by hybrid capture using the Agilent_MUG_Solid_DNAV6 panel, covering approximately 800 genes (~3.5 Mb; Agilent Technologies, Santa Clara, CA, USA), and sequenced on a DNBSEQ‐T7 sequencer (Wuhan MGI Tech Co., Ltd., Wuhan, Hubei, China). Variants were identified using the institutional bioinformatics pipeline. Microarray‐based copy‐number analysis (Illumina, San Diego, CA, USA; Sentrix identifiers: 209503260096_R08C01 and 209503260096_R07C01) was additionally performed, and the resulting data were evaluated using the EpiDiP Methylome & CNV Viewer (J. Hench, IfP Basel, Basel, Switzerland, version 8.0c; https://epidip.org/sh/apps/allMethylomesCNV08c/; accessed February 2026).

### Clinical data analysis

Clinical records from involved specialties were reviewed to obtain data on localization, surgical approach, date of diagnosis, recurrence, last follow‐up, age, and sex.

### Statistical analyses

Descriptive statistics were computed using IBM SPSS Statistics, Version 25 (IBM Corp., Armonk, NY, USA). Continuous variables are reported as means, medians, and ranges, while categorical variables are presented as absolute counts and percentages. Given the descriptive and exploratory nature of the study and the limited sample sizes in subgroups, no formal assessment of data distribution or inferential statistical analyses were performed.

## Results

### Clinical and molecular characteristics

The cohort comprised 283 cases of NF. Patients' age ranged from 1 to 64 years, with a median age of 31.5 years at diagnosis. There was a near‐equal sex distribution, with 143 females and 140 males. A second search of the database, specifically looking at the *USP6*‐rearranged mesenchymal neoplasms, showed 56 cases. A total of 56 *USP6*‐rearranged mesenchymal neoplasms, comprising 44 NF (78.6%; Table 1), 8 ABCs (14.3%), 2 MO (3.6%), 1 FO (1.8%), and 1 PM‐like lesion (1.8%) were identified (supplementary material, Table S2).

**Table 1:** Clinical characteristics of the reviewed NF cases

Variable	NF, *n* = 44
Age, years	Median 31.5 (range 1–64)
Sex	22 M/22 F
Lesion size*	Median 1.8 cm (range 0.8–5 cm); measured in 18/44
Anatomic region	Trunk: 12 (27.3%) Upper ext.: 12 (27.3%) Head/neck: 10 (22.7%) Lower ext.: 9 (20.5%) NA: 1 (2.3%)
Behavior	No recurrences or aggressive clinical course observed

F, female; M, male; NF, nodular fasciitis.

* Lesion size represents maximum dimension; only cases with documented measurements were included.

### Nodular fasciitis

NF patients ranged in age from 1 to 64 years (median: 31.5 years), with an almost equal sex distribution (22 males and 22 females) (Table 1). Lesions most involved the trunk region (12; 27.3%), upper extremity (12; 27.3%), followed by the head and neck region (10; 22.7%) and lower extremity (9; 20.5%). The median lesion size was 1.8 cm (range: 0.8–5 cm). NF demonstrated the greatest diversity of *USP6* fusion partners, with 18 distinct partner genes identified among 44 cases. *MYH9*::*USP6* was the most frequent fusion (18/44; 40.9%), followed by *COL1A1*::*USP6* (5/44; 11.4%), *MIR22HG*::*USP6* (3/44; 6.8%), and *RRBP1*::*USP6* (3/44; 6.8%). *ASPN*::*USP6* was identified in two cases (2/44; 4.5%), whereas the remaining 13 fusion partners were each detected in single cases (Figures 1, 2; supplementary material, Figure S1).

**Figure 1:**
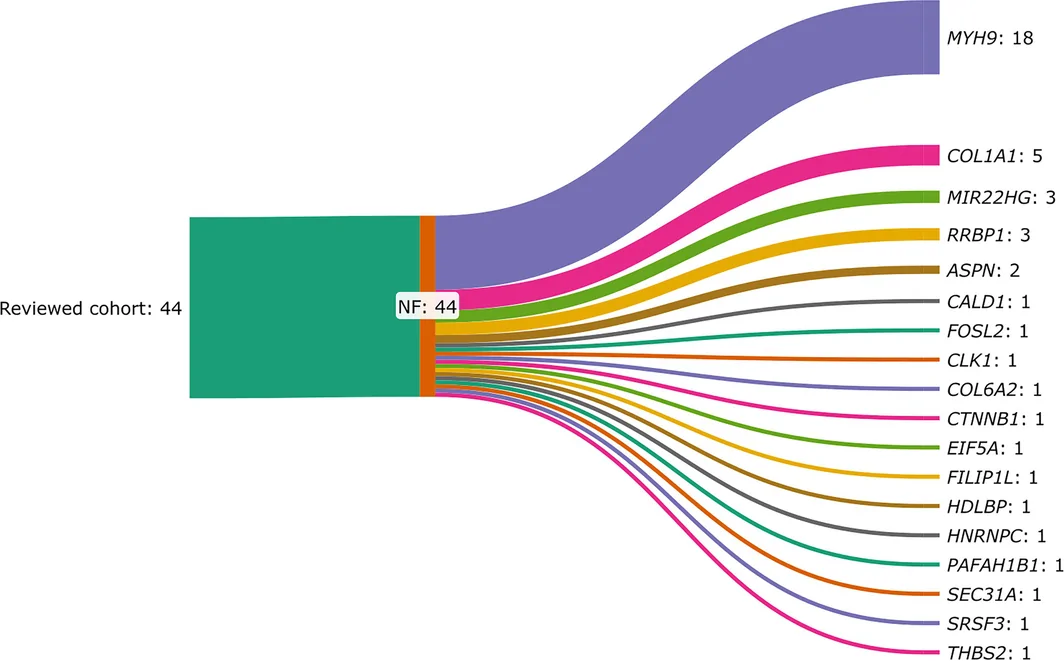
Sankey visualization of USP6 fusion partners in nodular fasciitis. The Sankey diagram summarizes the distribution of USP6 fusion partners among the 44 molecularly confirmed nodular fasciitis cases included in the study. The width of each connection corresponds to the number of cases harboring the respective fusion partner. *MYH9* was the most frequent fusion partner.

**Figure 2:**
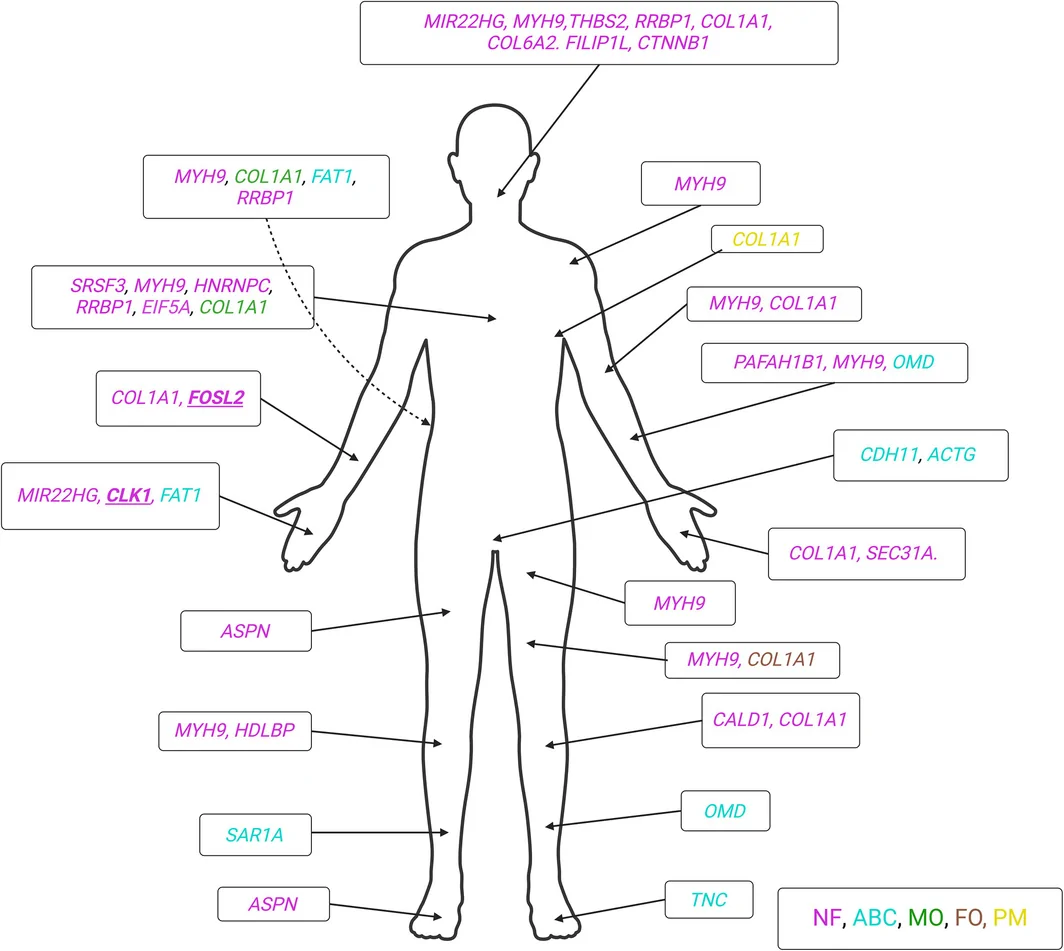
Anatomical distribution of recurrent gene fusions in *USP6*‐associated lesions. Schematic illustration of the anatomical distribution of our reported *USP6* fusion partners across the body. Labelled boxes indicate recurrent fusion partner genes reported at the respective anatomical sites, with arrows indicating approximate lesion locations. Gene names are color‐coded according to tumor entity: nodular fasciitis (NF, magenta), aneurysmal bone cyst (ABC, cyan), myositis ossificans (MO, green), fasciitis ossificans (FO, brown), and proliferative myositis‐like lesion and related lesions (PM, yellow). Genes shown in multiple colors indicate occurrence in more than one lesion type. Created with BioRender.com. Skok, K. (2026). https://BioRender.com/jh3vu7y.

Importantly, several fusion partners identified in this cohort, including *CLK1*, *FILIP1L*, and *HNRNPC*, are extremely rare in NF. Morphologically, all tumors (except specifically described cases) displayed a classic NF morphology with consistently strong SMA expression in a tram track‐like fashion. Cases with classic NF morphology and focal infiltrative growth that did not recur are not individually described. Regarding NF with, to the best of our knowledge, not yet reported fusion partners (*CLK1*::*USP6, FOSL2*::*USP6*), we have carefully examined these cases and report the individual findings (Table 2).

**Table 2:** Immunohistochemical profile of the cases with infiltrative, aggressive‐appearing morphology

Stain	Case 1	Case 2
SMA	+++	+++
CD34	Focal +	−
Desmin	−	−
KI‐67	~10%	~10%

+, positive; −, negative.

### Cases with ‘novel’ fusion partners

#### Case 1: *CLK1::USP6* Fusion

A 1‐year‐old male presented with a soft‐tissue lesion of the palm (Figure 3). Histologic examination revealed multiple fragments of soft tissue composed of spindle‐shaped cells with elongated oval to focally round nuclei, arranged in short fascicles in a collagenous background. Immunohistochemically, the lesional cells showed diffuse strong SMA positivity. The tumor cells were negative for desmin, SOX10, pan‐TRK, myogenin (clone MyoFD5), and MyoD1. CD34 was negative in the tumor cells and highlighted vascular structures. Internal and on‐slide controls showed appropriate staining performance. Molecular analysis demonstrated the presence of a *CLK1*::*USP6* fusion.

**Figure 3:**
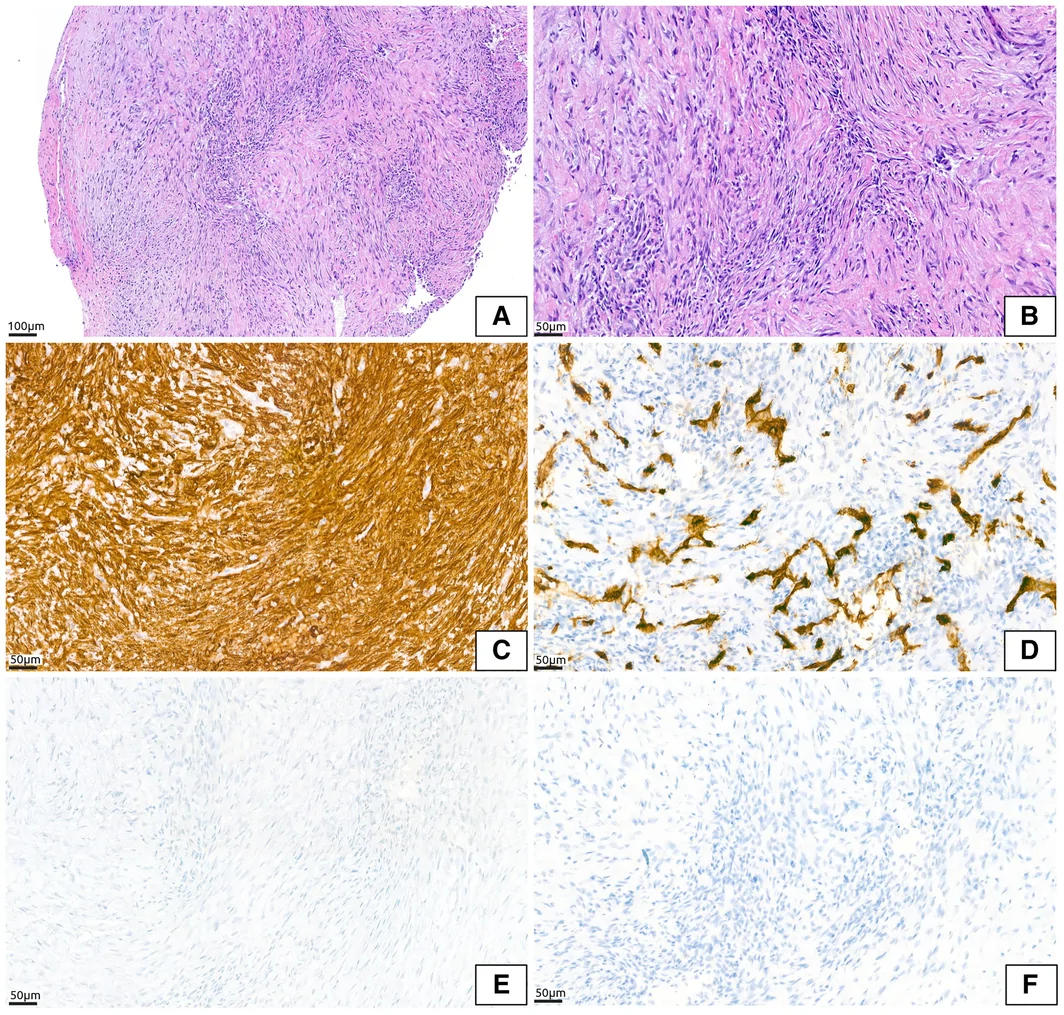
Nodular fasciitis with *CLK1*::*USP6* Fusion in a 1‐year‐old male. (A) Low‐power overview (H&E, 10×) showing a moderately cellular spindle‐cell proliferation with a fascicular growth pattern. (B) Higher magnification (H&E, 40×) highlighting short fascicles of bland spindle‐shaped cells with elongated to focally oval nuclei embedded in a predominantly collagenous stroma, without significant cytologic atypia. (C) Immunohistochemistry demonstrating diffuse and strong cytoplasmic positivity for smooth muscle actin (SMA) in the lesional cells (20×). (D) CD34 staining demonstrates the rich vascularization of the lesion (20×). (E–F) Negative immunostaining for desmin and myogenin in the lesion (20×). Scale bars (black lines) vary between panels (different magnifications); their values are indicated in the respective images. Compiled in BioRender. Skok, K. (2026). https://BioRender.com/smabknx.

#### Case 2: *FOSL2::USP6* fusion

A 31‐year‐old female presented with a soft‐tissue lesion of the upper extremity (arm). Histologic examination (Figure 4) revealed fibrous and adipose tissue with an infiltrative spindle cell proliferation, arranged in fascicles and embedded in a predominantly myxoid stroma. The spindle cells showed round to elongated oval nuclei with bipolar basophilic cytoplasmic processes. Cellular density was variable, ranging from moderately to highly cellular areas. A markedly increased mitotic activity was observed, with up to 15 mitoses per 10 high‐power fields, while atypical mitotic figures were not identified. The lesion was traversed by a delicate capillary vascular network, and the spindle cells showed infiltration between adjacent adipocytes. Immunohistochemically, the lesional cells demonstrated diffuse SMA positivity, while the rest of the performed IHC stains remained negative, and H3K27me3 retained. Molecular analysis identified a *FOSL2*::*USP6* fusion.

**Figure 4:**
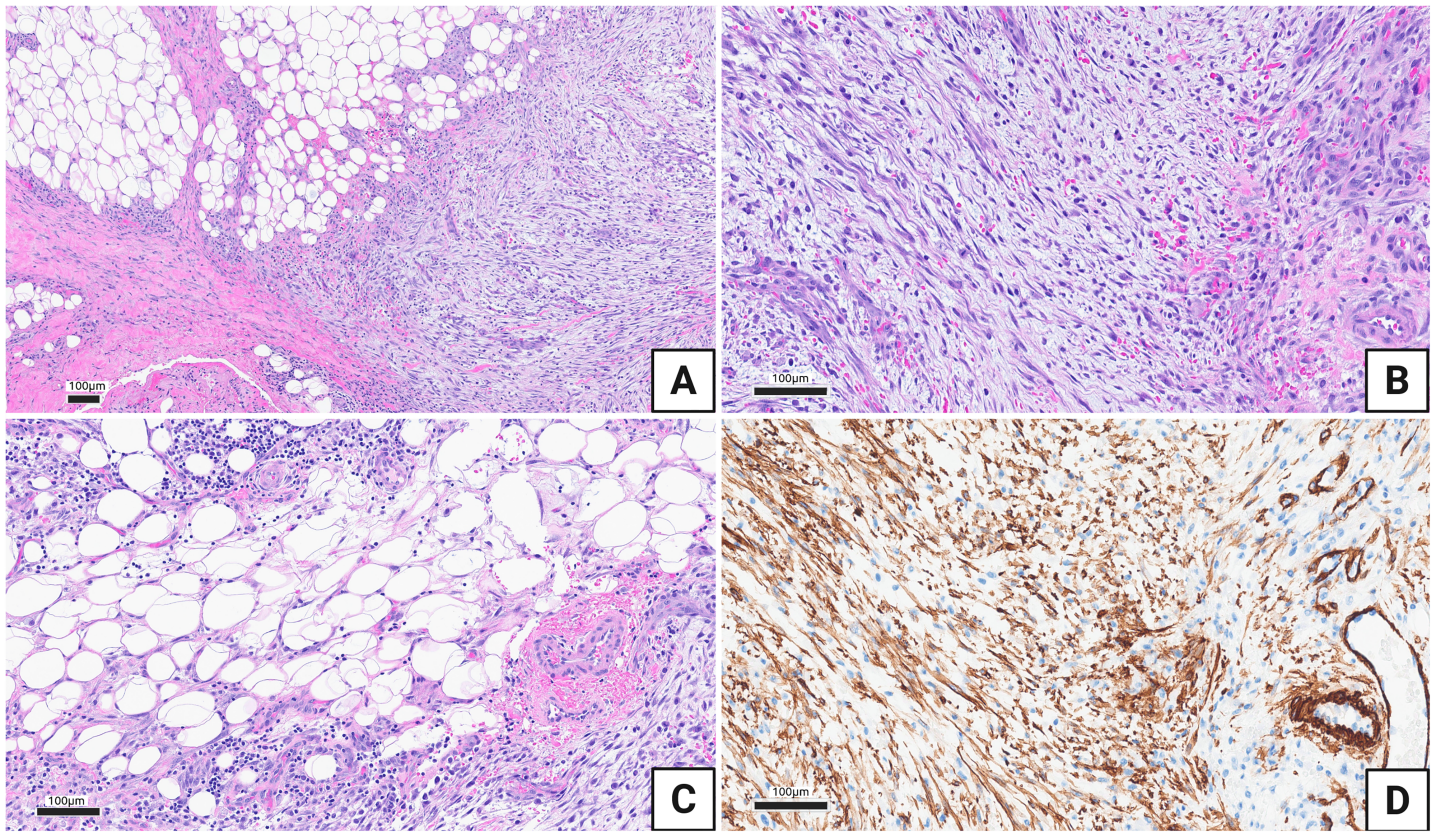
Nodular fasciitis with *FOSL2*::*USP6* fusion in a 31‐year‐old female. (A) Low‐power view showing a poorly circumscribed spindle cell proliferation infiltrating surrounding adipose tissue (10×, H&E). (B) Higher magnification demonstrates a moderately cellular proliferation of bland spindle‐shaped cells arranged in short fascicles and a predominantly myxoid stroma with delicate vessels (20×, H&E). (C) Infiltrative growth pattern with entrapment of adipocytes and adjacent structures (20×, H&E). (D) Immunohistochemical staining for SMA highlights the lesional spindle cells from Panel B (20×). Scale bars (black lines) vary between panels (different magnifications); their values are indicated in the respective images. Compiled in BioRender. Skok, K. (2026). https://BioRender.com/oqmq9em.

### Cases with infiltrative, aggressive‐appearing morphology (class 3 – see Discussion) show no additional genomic complexity

#### Case 1 – Female, 41 years, upper leg, *ASPN* :: *USP6*

The HE sections reveal a spindle‐cell mesenchymal neoplasm with a distinctly diffuse infiltrative growth pattern into the surrounding soft tissues. In areas, the tumor exhibits morphological features reminiscent of fibromatosis, with fascicles of spindle cells and elongated, compressed blood vessels. The proliferation diffusely infiltrates adipose tissue (Figure 5A,B). Focally, the tumor shows increased cellularity with mildly enlarged nuclei and a conspicuous rise in mitotic figures. Small foci of metaplastic bone are present (Figure 5C). Scattered extravasated erythrocytes are identified (Figure 5D).

**Figure 5:**
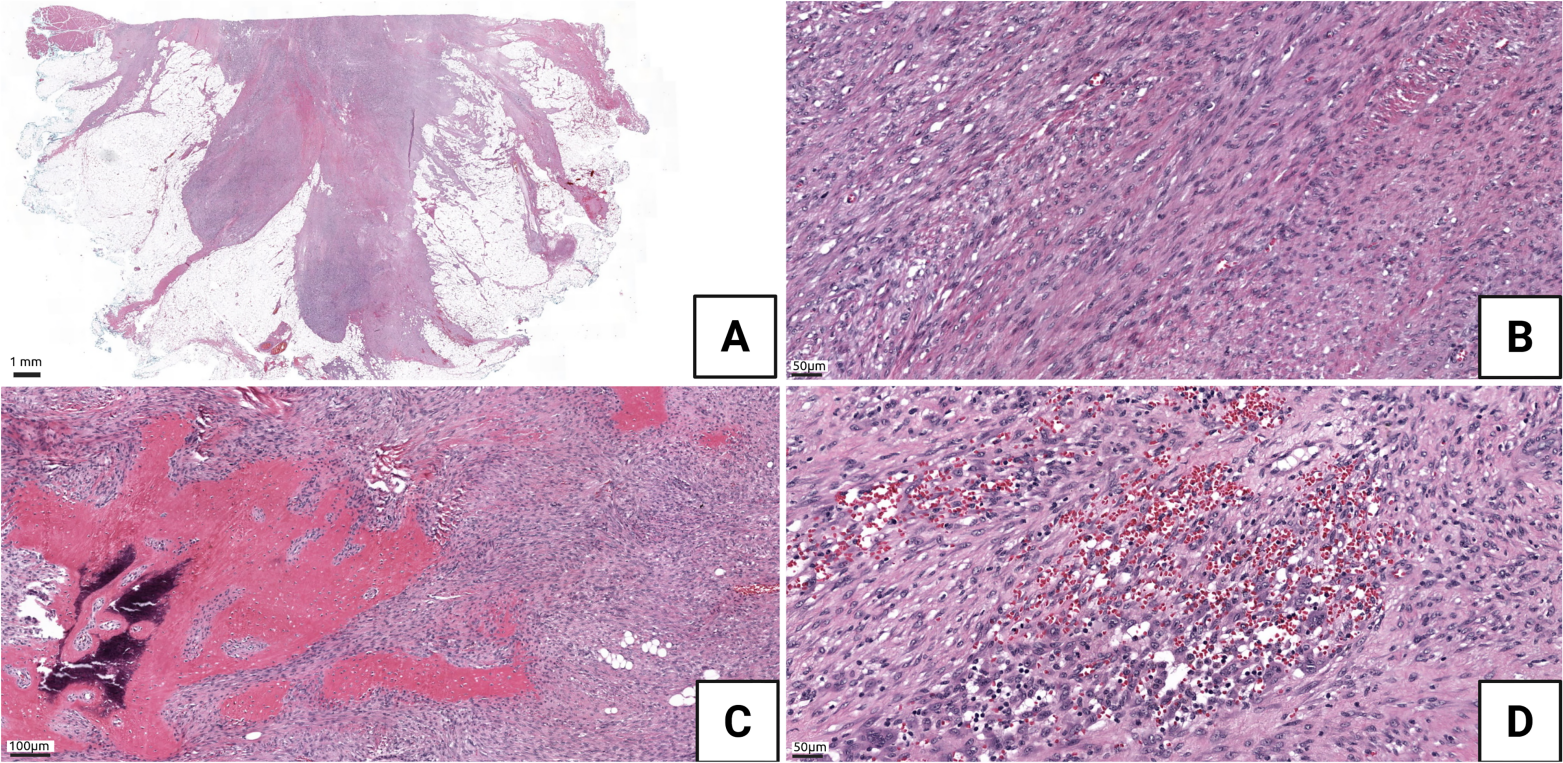
Infiltrative nodular fasciitis with aggressive‐appearing morphology (case 1). (A) Low‐power overview showing a spindle‐cell mesenchymal proliferation infiltrating the surrounding soft tissue. (B) Long fascicles of spindle cells similar to fibromatosis (40×). (C) Foci of metaplastic bone formation (20×). (D) Extravasated erythrocytes and occasional multinucleated giant cells (40×). Scale bars (black lines) vary between panels (different magnifications); their values are indicated in the respective images. Compiled in BioRender. Skok, K. (2026). https://BioRender.com/kqzdp6j.

#### Case 2 – Male, 34 years, thorax, *RRBP1* :: *USP6*

The HE sections reveal a spindle‐cell mesenchymal neoplasm with a distinctly diffuse infiltrative growth pattern with prominent infiltration into skeletal muscle and infiltration of adipose tissue (Figure 6A,B). At the periphery an unusual multinodular growth pattern (Figure 6C) and a rich vascular network composed of medium‐sized vessels is seen (Figure 6D,E). The spindle cells are arranged in long fascicles with compressed vascular structures (Figure 6F). Scattered extravasated erythrocytes and inflammatory cells mainly composed of lymphocytes and plasma cells are present (Figure 6G) as well as focal osteoclastic giant cells (Figure 6H). Most spindle cells have elongated, ovoid nuclei with vesicular chromatin and small nucleoli. The cytoplasm is indistinct. No markedly increased mitotic activity is noted (1–2 mitoses/10 high‐power fields).

**Figure 6:**
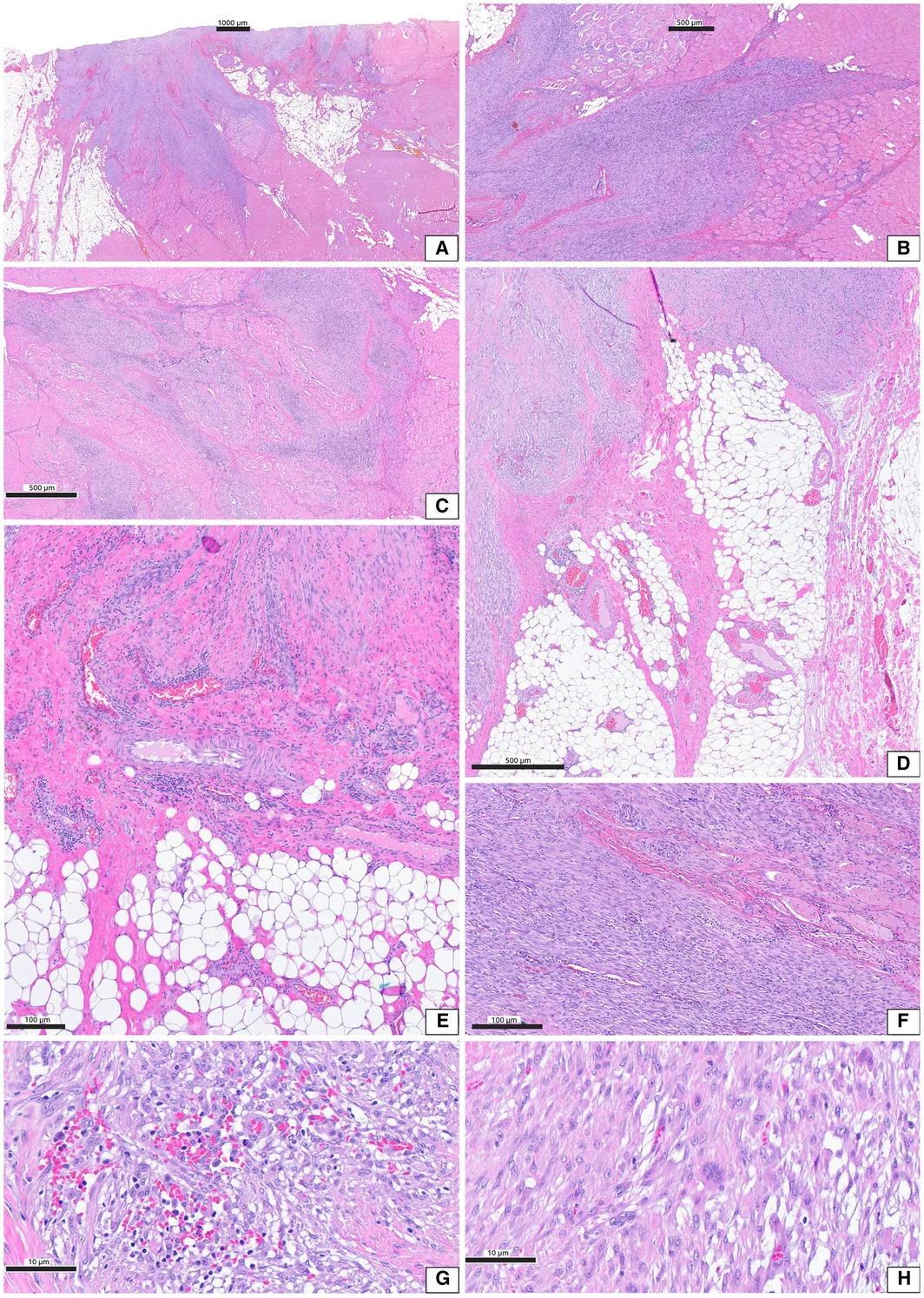
Infiltrative nodular fasciitis with aggressive‐appearing morphology (case 2). (A) Low‐power overview (H&E) demonstrating an infiltrative growth pattern extending into adjacent skeletal muscle and adipose tissue. (B) Infiltration of skeletal muscle by fascicles of spindle cells. (C) Multinodular growth pattern at the lesion periphery. (D, E) Rich vascular network, including medium‐sized vessels, predominantly at the lesion periphery. (F) Long, sweeping fascicles of spindle cells associated with a prominent inflammatory infiltrate. (G) Areas with extravasated erythrocytes. (H) Intermingled multinucleated giant cells within the spindle‐cell proliferation. Scale bars (black lines) vary between panels (different magnifications); their values are indicated in the respective images. Compiled in BioRender. Skok, K. (2026). https://BioRender.com/ewijfvl.

### Molecular diagnostics from both cases

Detection of *USP6* rearrangements was successfully achieved in both cases using the Archer® FusionPlex Sarcoma v4 (Diagnostic and Research Institute of Pathology) (in‐house kit). Case 1 (*ASPN*::*USP6* fusion) demonstrated a high fusion abundance, with 95.1% of *USP6*‐aligned reads supporting the fusion event (969 reads; 138 unique start sites). In contrast, Case 2 showed an *RRBP1*::*USP6* fusion with 18.7% fusion‐positive *USP6* reads (72 reads; 40 start sites), correlating with its substantially better DNA integrity. Subsequent hybrid‐capture DNA sequencing (≈3.5 Mb panel, 800 genes) identified no pathogenic somatic mutations in either case. Likewise, copy‐number variation (CNV) analysis revealed no chromosomal gains or losses indicative of structural genomic instability. Overall, both lesions exhibited a genetically stable diploid profile.

### DNA methylation‐based clustering consistent with NF

Both cases underwent DNA methylation microarray analysis, from which copy‐number profiles and unsupervised methylation‐based embeddings were derived [16]. In both profiles, no significant copy‐number gains or losses were detected, apart from minor stochastic variations considered within normal analytical noise. Unsupervised clustering positioned both cases firmly within the NF molecular reference region, closely aligning with multiple canonical *USP6*‐rearranged NF cases in the database.

### Non‐NF USP6‐associated entities

In contrast to NF, the non‐NF *USP6*‐associated lesions demonstrated a more restricted fusion spectrum. ABC (*n* = 8) occurred exclusively in osseous sites and harbored *FAT1*::*USP6* (*n* = 2), *OMD*::*USP6* (*n* = 2), and single cases with *ACTG1*::*USP6*, *CDH11*::*USP6*, *SAR1A*::*USP6*, and *TNC*::*USP6*. MO (*n* = 2) showed *COL1A1*::*USP6* in both cases. FO (*n* = 1) and PM‐like lesion (*n* = 1) also revealed *COL1A1*::*USP6* fusions. No aggressive recurrences or metastatic behavior were observed in any of these lesions during the available follow‐up.

## Discussion

The present study expands the clinicopathological and molecular understanding of NF while providing descriptive context regarding other *USP6*‐associated lesions encountered during the database review. A key aspect of the study is the detailed morphologic and genomic characterization of selected NF cases with distinctly infiltrative, ‘aggressive‐appearing’ morphology.

Our findings support three principal observations. First, the two NF cases with distinctly infiltrative and otherwise alarming morphology remained genomically simple and clinically indolent following comprehensive molecular characterization. Second, the molecularly investigated cohort demonstrated substantial diversity of *USP6* fusion partners, including rare and previously unreported fusions. Third, the broader retrospective cohort confirmed the predominantly benign clinical behavior of NF.

The following four‐class framework should be regarded as an exploratory conceptual model for the morphologic categorization of *USP6*‐rearranged NF. It is intended to organize the clinicopathologic spectrum observed in the present cohort and reported in the literature rather than to define biologically distinct entities. The proposed subclasses integrate recurring morphologic patterns with currently reported genetic alterations that may provide additional biological context and inform future refinement of this framework following validation in larger, independent cohorts (Table 3).

**Table 3:** Proposed conceptual framework categorizing *USP6*‐rearranged nodular fasciitis into four classes based on morphologic features, biological behavior, and molecular alterations

Class	Histomorphology	Growth pattern/Local behavior	Cytologic atypia	Molecular features	Clinical course/Biological behavior	Typical examples/notes
Class 1 – Conventional NF	Classic NF morphology (plump myofibroblasts, tissue culture–like appearance, myxoid to collagenous stroma, extravasated RBCs)	Usually circumscribed; may appear rapidly growing during proliferative phase	Absent	*USP6* fusion (various partners such as *MYH9*::*USP6*, *SPTAN1*::*USP6*)	Benign, self‐limited; occasional recurrence during proliferative phase	Unusual anatomical location or a clinically alarming presentation should prompt molecular confirmation of a USP6 rearrangement
Class 2 – Conventional NF associated with reported aggressive clinical behavior	Conventional NF morphology	Infiltrative growth, progressive erosion of the adjacent bone	Absent	*USP6* fusion; additional genomic alterations have been reported in selected cases (e.g. amplification of *PPP6R3* and *USP6*)	Unexpectedly aggressive course with recurrence and rare metastasis	Aggressive clinical behavior has been suggested with additional amplifications. Detection of certain fusion partners or amplifications may confer aggressive phenotype and requires molecular work‐up and follow‐up. Rare, reported cases; biological significance remains uncertain.
Class 3 – Infiltrative NF with indolent clinical behavior	NF morphology but with worrisome architectural features	Infiltrative margins, multinodularity, vascular proliferation, increased mitotic activity	No overt cytologic atypia	*USP6* fusion; otherwise genomically stable profile (no significant CNV, mutation burden, or methylation deviation)	Indolent clinical course despite alarming morphology	Includes the present study cases and previously reported cases. Molecular profiling supports classification within the NF spectrum despite infiltrative architecture
Class 4 – Cytologically atypical/sarcoma‐like *USP6*‐rearranged lesions	NF architecture (circumscribed) but marked cytologic atypia (pleomorphism, atypical mitoses)	Infiltrative growth pattern rarely described, histologically alarming	Present (pleomorphism, atypical mitoses)	*USP6* fusions with various partners (e.g. *CALD1*::*USP6*, *PAFAH1B1*::*USP6*, *COL6A2*::*USP6*, *EIF5A*::*USP6*); genetic and epigenetic profile consistent with nodular fasciitis. Data lacking/limited on additional genetic changes	Malignant behavior (distant metastases) not proven on follow up, but lesions may mimic sarcoma	Rare subset; particularly described in pediatric or unusual sites; risk of overtreatment as high‐grade Sarcoma

CNV, copy number variations; TMB, tumor mutational burden.

Class 1 comprises morphologically conventional NF, which represents the predominant clinicopathological phenotype and is characterized by classic histomorphology and typically benign, self‐limited biological behavior. In the vast majority of cases, the combination of characteristic morphology and an appropriate clinical context is sufficient for diagnosis. A small subset of otherwise conventional lesions may, however, present with clinically alarming features, including rapid enlargement, pain, neurological symptoms, site‐related functional impairment, or, rarely, early local recurrence [17, 18, 19, 20, 21, 22]. The diagnostic challenges described in the literature include lesions arising at uncommon or anatomically sensitive sites, such as the periorbital soft tissues, a peripheral nerve, the joint synovium, the buccal mucosa, and the palm, where NF may clinically or radiologically mimic a malignant neoplasm [17, 18, 19, 20, 21]. In addition, rapidly enlarging lesions may produce substantial local morbidity despite entirely conventional histomorphology, as illustrated by the palmar NF associated with nerve‐compression symptoms reported by Sawamura *et al* [21]. Rare recurrence after apparently complete excision has also been documented in oral NF [22]; incomplete removal and persistence of an actively proliferating lesion may plausibly contribute to recurrence. Overall, these presentations are more likely to reflect the dynamic growth characteristics, anatomical setting, and local effects of NF than true biological progression. In clinicopathologically discordant or otherwise challenging cases, demonstration of a USP6 rearrangement in combination with classic morphology may therefore increase diagnostic confidence, facilitate appropriate classification, distinguish conventional NF from malignant or site‐specific mimics, and reduce the risk of unnecessary aggressive treatment [17, 18, 19, 20, 21, 22].

Class 2 comprises NF with entirely conventional histomorphology but potential for aggressive biological behavior. This concept is based on a very limited number of reported cases in which morphologically typical NF exhibited an unexpected clinical course characterized by infiltrative growth, progressive erosion of the adjacent bone, repeated local recurrence, or, exceptionally, distant metastasis [14, 23, 24]. Two independent reports identified tumors harboring a *PPP6R3*::*USP6* fusion [23, 24], while Saoud *et al* additionally described aggressive NF with a *COL1A1*::*USP6* fusion and one further case with a *PPP6R3*::*USP6* fusion [14]. In the index case reported by Guo *et al*, the unusual clinical behavior was accompanied by amplification of both *PPP6R3* and *USP6*, resulting in increased *USP6* expression, suggesting that secondary genomic alterations, rather than the fusion partner alone, may contribute to the altered biological phenotype [23]. However, comprehensive genomic profiling was unavailable in most reported cases [14, 24], and the current evidence is insufficient to establish a causal or prognostic association between specific *USP6* fusion partners and aggressive behavior. Nevertheless, morphologically conventional NF presenting with an unusually deep‐seated location, infiltrative growth, progressive bone erosion, repeated recurrence, or metastatic disease should prompt comprehensive molecular characterization extending beyond fusion detection and warrant close clinical follow‐up to further define this rare clinicopathological subset.

Class 3 comprises NF with an unusual infiltrative growth pattern. These lesions are characterized by distinctly infiltrative margins partly in combination with multinodular architecture, prominent vascular proliferation, and variable mitotic activity while lacking overt cytologic atypia or atypical mitotic figures. The two cases in our series exemplify this pattern and both followed an indolent clinical course. We showed that despite their worrisome morphology, molecular profiling including mutation analysis, copy‐number assessment, and DNA methylation profiling, demonstrated a genomically stable profile beyond the USP6 rearrangement. Furthermore, methylation profiling clustered both tumors with conventional NF, whereas low‐grade myofibroblastic sarcoma (LGMS) occupied distinct regions of the methylation embedding (https://epidip.org/; Methylome & CNV Viewer, J. Hench, IfP Basel, version 8.0c; 02/2026), supporting classification of these lesions within the spectrum of *USP6*‐rearranged NF.

Likewise, Malik *et al* [25] reported three deep‐seated pediatric myofibroblastic tumors harboring *COL1A1*::*USP6* or *COL3A1*::*USP6* fusions that displayed infiltrative growth and clinically alarming features but lacked overt malignant cytology and behaved benignly following conservative excision [25]. Wang *et al* [26] further described a large intramuscular NF with infiltrative borders, increased mitotic activity (not more than 10 mitoses/HPF), and pseudosarcomatous morphology that raised suspicion for fibrosarcoma; however, demonstration of a *USP6* rearrangement confirmed the diagnosis, and no recurrence or metastasis developed during 56 months of follow‐up [26]. Collectively, these observations indicate that infiltrative growth, increased mitotic activity, deep location, or locally aggressive clinical and radiological features do not necessarily predict aggressive biological behavior in *USP6*‐rearranged NF. In such diagnostically challenging cases expanding the molecular profile of these rare but morphologically distinctive tumors will improve classification and distinction between class 2 and 3.

Class 4 includes *USP6*‐rearranged lesions with frankly atypical or sarcoma‐like cytologic features (e.g. pleomorphism, marked nuclear atypia, atypical mitoses) but without proven malignant biological behavior [27, 28, 29, 30, 31, 32] or any specific genetic change. A small subset of these *USP6*‐rearranged lesions may in addition show infiltrative growth. Pediatric cases of NF with strikingly atypical or overtly malignant‐appearing morphology have been reported, including lesions with marked pleomorphism, increased cellularity, atypical mitotic figures, ulceration, and other features that may closely mimic sarcoma. Despite these concerning histologic findings, recurrent *USP6* rearrangements (e.g. *CALD1*::*USP6, PAFAH1B1*::*USP6, COL6A2*::*USP6*, and *EIF5A*::*USP6*) support their interpretation as part of the spectrum of *USP6*‐associated lesions and underscore the potential risk of overtreatment.

With respect to the morphomolecular distribution of USP6 fusion partners and their apparent anatomical tropism, our findings partially corroborate those of Cordier *et al* [12]. Both cohorts demonstrate considerable molecular heterogeneity, with multiple fusion partners occurring across different anatomical sites. *MYH9*::*USP6* was the most consistently shared fusion, occurring in nearly all anatomical regions in both studies. Additional overlapping fusion partners included *COL1A1* in the hand, *COL6A1* and *CTNNB1* in the head and neck region, *ASPN* in the thigh, and *PAFAH1B1* in the arm. The trunk and head and neck region exhibited the greatest diversity of fusion partners in our cohort, whereas the extremities showed less overlap and a higher proportion of unique fusions. The trunk shared only *MYH9*::*USP6* between both cohorts, while the head and neck region additionally shared *CTNNB1*::*USP6* and *COL6A2*::*USP6*. Furthermore, *MYH9*::*USP6* was identified in upper‐leg lesions in our cohort but not in the series reported by Cordier *et al* [12] (Figure 2). The reasons for these regional differences remain unclear. They may reflect biological variation related to the greater anatomical and mesenchymal diversity of the trunk and head and neck region, but sampling effects cannot be excluded. Beyond NF, our cohort also included other *USP6*‐associated neoplasms, including MO, FOP, ABC, and PM‐like lesion. Among these entities, *COL1A1*::*USP6* was identified in FOP, MO, PM‐, and NF, making it the only fusion partner shared across multiple tumor types as well as between both cohorts. Although PM has historically been considered closely related to NF, recent molecular findings suggest a more heterogeneous pathogenesis (*FOS* rearrangement). In this context, the *COL1A1::USP6*‐positive PM‐like lesion in our cohort may further illustrate the morphological overlap within the expanding spectrum of *USP6*‐rearranged lesions.

Based on our findings and the available literature, additional molecular characterization beyond *USP6* fusion detection should be considered for NF‐like lesions with infiltrative growth mimicking low‐grade myofibroblastic sarcoma (class 3), lesions with worrisome sarcoma‐like cytologic features (class 4), recurrent tumors, cases with discordant clinicoradiologic and histopathologic findings irrespective of morphologic class, and lesions harboring rare *USP6* fusion partners that have been reported in association with aggressive clinical behavior (class 2). Such analyses may help identify additional genomic alterations that contribute to unusual infiltrative growth, marked cytologic atypia, or exceptional clinical behavior. Although the available evidence remains limited, the rare, reported cases with aggressive biological behavior have also demonstrated additional genomic alterations beyond the primary *USP6* rearrangement, suggesting that secondary molecular events may contribute to biological progression in a subset of *USP6*‐associated neoplasms. Additional well‐characterized cases with comprehensive genomic profiling and long‐term clinical follow‐up will be essential to refine risk stratification, better define biological boundaries within the spectrum of *USP6*‐associated neoplasms, and inform future iterations of tumor classification systems. Based on the currently available clinicopathological and molecular evidence, we propose that these lesions should remain within the spectrum of *USP6*‐associated neoplasms despite their morphologic overlap with low‐grade myofibroblastic sarcoma (Table 4).

**Table 4:** List of published difficult and/or morphologically equivocal NF cases

Study	Age (years)	Sex/Male to female ratio	Site	Size (cm)	Nuclear pleomorphism/Necrosis	MI (/HPFs)	Atypical mitotic figures	Gene fusions	Treatment	LR/DM	Status at last FU	FU (months)	Study type
Qiu *et al* [29]	1,8	M	Left scapular area	2.6	No/No	10/10	Yes	*PAFAH1B1*::*USP6*	Resection	No	NED	39	Case report
Tomassen *et al* [28]	10	M	Chest wall	2.4	Yes/No	Numerous	Yes	*COL6A2*::*USP6*	Resection	No	NED	22	Case report
Lenz *et al* [32]	41	F	Forearm	2–3	No/No	5/5	Yes	*EIF5A*::*USP6*	Resection	NA	NA	NA	Case report
Papke *et al* [30]	7	M	Dorsal wrist	2.2	Yes/No	7/10 t	Yes	*CALD1*::*USP6*	Resection	NA	NA	NA	Case report
Teramura *et al* [24]	27	M	Upper arm	6.3	Yes/Yes	5/10 t	NA	*PPP6R3*::*USP6*	Resection	No	NED	12	Case report
Arcovito *et al* [27]	56	M	Shoulder	NA	No/No	Increased	Yes	*THBS2*::*USP6*	Resection	No	NED	34	Case series
Arcovito *et al* [27]	29	F	Thigh	NA	No/No	Increased	Yes	*RRBP1*::*USP6*	Resection	No	NED	42	Case series
Arcovito *et al* [27]	58	M	Thigh	NA	No/No	Increased	Yes	*Unknown*::*USP6*	Resection	No	NED	38	Case series
Saoud *et al* [14]	Median: 36; Range: 15–59	4 M:5F	Forearm = 3; Thigh = 2; Shoulder = 1; Abdominal wall = 1; Chest wall = 1	Median: 2.2; range: 1.4–24	No/1	NA	No	*EIF4A1*::*USP6; FILIP1L::USP6; NF1*::*USP6; OMD*::*USP6; PFN1*::*USP6; RLIM*::*USP6; SETD5*::*USP6; PPP6R3*::*USP6; COL1A1*::*USP6*	Resection = 6; Observation = 3	1/1	Died of disease = 1; NED = 4; Alive with disease = 1	NA	Case series
Saoud *et al* [14]	51	M	Abdominal wall	2.2	NA	1.1	No	*FILIP1L*::*USP6*	Observation	No	NED	9	Part of case series
Saoud *et al* [14]	43	F	Chest wall	1.4	NA	3.1	No	*PFN1*::*USP6*	Resection	No	NED	10	Part of case series
Saoud *et al* [14]	34	F	Forearm	2	NA	NA	No	*RLIM*::*USP6*	Resection + margin	NA	NA	NA	Part of case series
Saoud *et al* [14]	22	F	Forearm	1.5	NA	1.1	No	*SETD5*::*USP6*	Resection	No	NED	10	Part of case series
Saoud *et al* [14]	36	M	Shoulder	NA	NA	NA	No	*NF1*::*USP6*	Resection	NA	NA	NA	Part of case series
Saoud *et al* [14]	13	F	Oral cavity	NA	NA	NA	No	*OMD*::*USP6*	Resection	NA	NA	NA	Part of case series
Saoud *et al* [14]	42	M	Forearm	3	NA	2.1	No	*EIF4A1*::*USP6*	Observation	No	NED	NA	Part of case series
Saoud *et al* [14]	59	F	Thigh/Hip	24	NA	<1/10	No	*PPP6R3*::*USP6*	Observation	NA	Alive with disease	108	Part of case series
Saoud *et al* [14]	36	M	Thigh	13.6	NA/1	15.1 t	No	*COL1A1*::*USP6*	Resection + margin	LR (14 months), M (lungs, 19 months)	Died of disease	22	Part of case series
Cordier *et al* [12]	Median: 37; Range: 2–78	M:F Ratio: 1:1.1	Head = 24; Neck = 5; Trunk = 36; Abdominal wall = 3; Perineum = 2; Extremities = 52; NA = 2	NA	NA	NA	NA	NA	NA	NA	NA	NA	Case series
Cloutier *et al* [33]	27	F	Breast	0.7	NA	4/10	NA	*MYH9*::*USP6*	Observation	NA	No interval growth	5	Case series
Cloutier *et al* [33]	15	F	Breast	0.5	NA	3/10	NA	*MYH9*::*USP6*	Excision (for fibroadenoma) + Observation	NA	NA	NA	Case series
Cloutier *et al* [33]	38	F	Breast	0.5	NA	2/10	NA	*NA*	Excision	NA	Self‐resolved, no recurrence	108	Case series
Cloutier *et al* [33]	39	F	Breast	1.1	NA	3/10	NA	*CTNNB1*::*USP6*	Excision + Observation	NA	No recurrence	24	Case series
Cloutier *et al* [33]	24	F	Breast	1.9	NA	2/10	NA	*NA*	Excision	NA	No recurrence	60	Case series
Cloutier *et al* [33]	34	F	Axoööa	0.9	NA	4/10	NA	*NA*	Observation	NA	NA	NA	Case series
Cloutier *et al* [33]	61	F	Breast	0.5	NA	12/10 t	NA	*NACA*::*USP6*	Excision	NA	No recurrence	67.2	Case series
Cloutier *et al* [33]	47	F	Breast	>1.9	NA	4/10	NA	*LDHA*::*USP6*	Observation	NA	Self‐resolved; no recurrence	67.2	Case series
Cloutier *et al* [33]	32	F	Breast	0.4	NA	1/10	NA	*MYH9*::*USP6*	Excision	NA	No recurrence	8	Case series
Cloutier *et al* [33]	31	F	Axilla	0.7	NA	7/10	NA	*SFLN11*::*USP6*	Observation	NA	Self‐resolved; no recurrence	33.6	Case series
Cloutier *et al* [33]	22	F	Breast	2.3	NA	2/10	NA	*MYH9*::*USP6*	Excision	NA	NA	NA	Case series
Cloutier *et al* [33]	26	F	Breast	5.8	NA	3/10	NA	*NA*	Excision	NA	NA	NA	Case series
Sawamura *et al* [21]	53	F	Hand	3	Yes/No	NA	Yes	*MYH9*::*USP6*	Marginal resection	No/No	NED	24	Case report
Dworak *et al* [17]	16	M	Periorbital	1.5	NA/No	NA	NA	*NA*::*USP6*	Excisional biopsy	No	NED	NA	Case report
Parkinson *et al* [18]	29	F	Intraneural Femoral Nerve	NA	NA	NA	NA	*CTNNB1*::*USP6*	Excision	NA	NA	NA	Case report
Vuong *et al* [20]	41	F	Buccal	1.1	NA	Ki‐67 <5%	NA	*SPTAN1*::*USP6*	excision	No	NED	23	Case report
Stražar *et al* [19]	48	M	Left Hip	3.2	NA/No	Inconspicuous mitotic activity	NA	*MYH9*::*USP6*	Arthroscopic excision	No	NED	12	Case series
Stražar *et al* [19]	38	M	Right shoulder	2.2	NA/Yes	Inconspicuous mitotic activity	NA	*COL1A1*::*USP6*	Arthroscopic excision	No	NED	55	Case series
Stražar *et al* [19]	25	F	Right hip	2	NA/Yes	Inconspicuous mitotic activity	NA	*MYH9*::*USP6*	Arthroscopic excision	No	NED	120	Case series
Wang *et al* [26]	27	M	Left upper arm	5	NA	8/10	NA	*NA*::*USP6*	Excision	No	NED	56	Case report
Sápi *et al* [4]	52	M	L. thigh	NA	NA	0.5/10	NA	*NA*::*USP6*	NA	NA	NA	NA	Case series
Sápi *et al* [4]	40	M	R. little finger	NA		0.5/10	NA	*MYH9*::*USP6*	NA	NA	NA	NA	Case series
Sápi *et al* [4]	28	F	Face	NA	NA	1/10	NA	*MYH9*::*USP6*	NA	NA	NA	NA	Case series
Sápi *et al* [4]	44	M	L. Arm	NA	NA	1/10	NA	*MYH9*::*USP6*	NA	NA	NA	NA	Case series
Sápi *et al* [4]	9	M	Chest	NA	NA	1/10	NA	*MYH9*::*USP6*	NA	NA	NA	NA	Case series
Sápi *et al* [4]	52	M	L. knee	NA	NA	1/10	NA	*NA*::*USP6*	NA	NA	NA	NA	Case series
Sápi *et al* [4]	21	F	Forehead	NA	NA	2/10	NA	*NA*::*USP6*	NA	NA	NA	NA	Case series
Sápi *et al* [4]	5	M	R. gluteal region	NA	NA	5/10	NA	*MYH9*::*USP6*	NA	NA	NA	NA	Case series
Sápi *et al* [4]	42	F	L. shoulder blade	NA	NA	3/10	NA	*MYH9*::*USP6*	NA	NA	NA	NA	Case series
Sápi *et al* [4]	42	F	Forehead	NA	NA	3/10	NA	*NA*::*USP6*	NA	NA	NA	NA	Case series
Sápi *et al* [4]	17	F	Neck	NA	NA	6/10	NA	*MYH9*::*USP6*	NA	NA	NA	NA	Case series
Sápi *et al* [4]	21	M	R. shoulder	NA	NA	4/10	NA	*MYH9*::*USP6*	NA	NA	NA	NA	Case series
Sápi *et al* [4]	6	M	R supraclavicular region	NA	NA	3/10	NA	*MYH9*::*USP6*	NA	NA	NA	NA	Case series
Sápi *et al* [4]	12	M	Face	NA	NA	5/10	NA	*MYH9*::*USP6*	NA	NA	NA	NA	Case series
Sápi *et al* [4]	25	M	L. forearm	NA	NA	10/10 t	NA	*NA*::*USP6*	NA	NA	NA	NA	Case series
Sápi *et al* [4]	40	F	R. forearm	NA	NA	11/10 t	NA	*NA*::*USP6*	NA	NA	NA	NA	Case series
Sápi *et al* [4]	33	M	R. forearm	NA	NA	18/10 t	NA	*MYH9*::*USP6*	NA	NA	NA	NA	Case series
Balko *et al* [7]	30–35	F	Nuchal area	NA	NA	NA	NA	*NA*::*USP6*	NA	NA	NA	NA	Case series
Balko *et al* [7]	30–35	M	Supraclavicular bilateral	NA	NA	NA	NA	*NA*::*USP6*	NA	NA	NA	NA	Case series
Balko *et al* [7]	25–30	F	Inguinal area	NA	NA	NA	NA	*MYH9*::*USP6*	NA	NA	NA	NA	Case series
Balko *et al* [7]	45–50	F	Skin	NA	NA	NA	NA	*MYH9*::*USP6*	NA	NA	NA	NA	Case series
Balko *et al* [7]	35–40	F	R. zygomatic area	NA	NA	NA	NA	*MYH9*::*USP6*	NA	NA	NA	NA	Case series
Balko *et al* [7]	35–40	M	R. elbow	NA	NA	NA	NA	*MYH9*::*USP6*	NA	NA	NA	NA	Case series
Balko *et al* [7]	60–65	M	R. Face	NA	NA	NA	NA	*MYH9*::*USP6*	NA	NA	NA	NA	Case series
Balko *et al* [7]	50–55	M	Supraclavicular left	NA	NA	NA	NA	*MYH9*::*USP6*	NA	NA	NA	NA	Case series
Balko *et al* [7]	15–20	F	R. shoulder	NA	NA	NA	NA	*MYH9*::*USP6*	NA	NA	NA	NA	Case series
Balko *et al* [7]	30–35	F	Head	NA	NA	NA	NA	*MYH9*::*USP6*	NA	NA	NA	NA	Case series
Balko *et al* [7]	30–35	M	L. forearm	NA	NA	NA	NA	*MYH9*::*USP6*	NA	NA	NA	NA	Case series
Balko *et al* [7]	40–45	F	L. inguinal area	NA	NA	NA	NA	*MYH9*::*USP6*	NA	NA	NA	NA	Case series
Balko *et al* [7]	30–35	M	Hand	NA	NA	NA	NA	*MYH9*::*USP6*	NA	NA	NA	NA	Case series
Balko *et al* [7]	25–30	F	Deep tibial area	NA	NA	NA	NA	*TMP4*::*USP6*	NA	NA	NA	NA	Case series
Balko *et al* [7]	okt.15	F	R. temporal area	NA	NA	NA	NA	*SPARC*::*USP6*	NA	NA	NA	NA	Case series
Balko *et al* [7]	15–20	F	L. forearm	NA	NA	NA	NA	*EIF5A*::*USP6*	NA	NA	NA	NA	Case series
Balko *et al* [7]	10–15	M	L. arm	NA	NA	NA	NA	*KIF1A*::*USP6*	NA	NA	NA	NA	Case series
Balko *et al* [7]	0–5	M	Corner of the mouth	NA	NA	NA	NA	*MIR22HG*::*USP6*	NA	NA	NA	NA	Case series
Balko *et al* [7]	15–20	F	R. arm	NA	NA	NA	NA	*COL1A2*::*USP6*	NA	NA	NA	NA	Case series
Ovechko *et al* [22]	21	F	Buccal	2.7	NA	36/2 mm^2^		*NA*::*USP6*	Excisional biopsy	Yes/No	Recurrence after 3 weeks; NED after 6 months after second excision	6 in total, 4 after second excision	Case report

FU, follow‐up; HPF, high‐power field; LR/DM, local recurrence/distant metastasis; MI, mitotic index; NA, not available; NED, no evidence of disease.

This study has several limitations. Molecular testing was performed according to routine diagnostic indications rather than systematically across the entire cohort. Consequently, the molecular subset was enriched for consultation cases and diagnostically challenging lesions and should not be regarded as representative of the full clinicopathological spectrum of NF. The proposed framework is therefore exploratory and requires validation in independently assembled cohorts. We believe that rather than a binary benign–malignant framework, these lesions warrant a workup in which integrated morphologic, clinical, and molecular assessment is critical for accurate diagnosis. Future studies will determine whether specific molecular alterations carry prognostic significance. Taken together, the proposed four‐class framework should be regarded as an exploratory conceptual model summarizing the clinicopathological spectrum of NF. Future multicenter studies with systematic molecular characterization are required to determine whether any of the proposed subclasses carry biological or clinical significance.

## Author contributions statement

KS and BL‐A contributed to the conceptualization of the study. MTB and BL‐A contributed to data curation and formal analysis. KS, MTB and BL‐A contributed to the investigation. BL‐A provided supervision and validation. KS and BL‐A contributed to visualization. KS, MTB and BL‐A wrote the original draft of the manuscript. KS, MTB, AMS, TG, AL, CV and BL‐A contributed to the review and editing of the manuscript.

## Supporting information


**Figure S1.** Distribution of *USP6* fusion partners across benign fibro‐osseous and fibro‐myofibroblastic lesions
**Table S1.** Distribution of nodular fasciitis (NF) cases included in the study and the molecularly investigated subset according to referral source
**Table S2.** Clinical characteristics of the remaining *USP6*‐associated lesions found during database review

## Data Availability

The data supporting the findings of this study are included in the article and its supplementary material. Additional patient‐level data are not publicly available due to patient confidentiality restrictions but may be obtained from the corresponding author upon reasonable request, subject to applicable institutional approvals.

## References

[1] WHO Classification of Tumors Editorial Board . Soft Tissue and Bone Tumours, Volume 3 (5th edn). International Agency for Research on Cancer: Lyon (France), 2020.

[2] Price EB Jr , Silliphant WM , Shuman R . Nodular fasciitis: a Clinicopathologic analysis of 65 cases. Am J Clin Pathol 1961; 35: 122–136. 10.1093/ajcp/35.2.122 13737962

[3] Georgantzoglou N , Linos K . An update on selected cutaneous (myo) fibroblastic mesenchymal tumors. Semin Diagn Pathol 2023; 40: 295–305. 10.1053/j.semdp.2023.04.018 PMC10602371 37150655

[4] Sápi Z , Lippai Z , Papp G , *et al*. Nodular fasciitis: a comprehensive, time‐correlated investigation of 17 cases. Mod Pathol 2021; 34: 2192–2199. 10.1038/s41379-021-00883-x PMC8592838 34381187

[5] Miyama A , Kuratsu S , Takenaka S , *et al*. Two case reports of intra‐articular nodular fasciitis of the knee confirmed by MYH9‐USP6 gene fusion expression. J Orthop Sci 2021; 26: 1138–1142. 10.1016/j.jos.2018.12.008 30611632

[6] Igrec J , Brčić I , Igrec R , *et al*. Intraarticular nodular fasciitis of the knee with MHY9‐USP6 fusion: a case report. Int J Surg Pathol 2020; 28: 672–677. 10.1177/1066896920908054 32089028

[7] Balko J , Stanek M , Krskova L , *et al*. Unusual fusion gene rearrangements in patients with nodular fasciitis: a study of rare and novel USP6 fusion partners with a review of the literature. J Clin Pathol 2024; 77: 411–416. 10.1136/jcp-2023-208768 PMC11103289 36828621

[8] Nakayama S , Nishio J , Aoki M , *et al*. Ubiquitin‐specific peptidase 6 (USP6)‐associated fibroblastic/myofibroblastic tumors: evolving concepts. Cancer Genomics Proteomics 2021; 18: 93–101. 10.21873/cgp.20244 PMC7943209 33608306

[9] Pižem J , Šekoranja D , Matjašič A , *et al*. The role of molecular diagnostics in aneurysmal and simple bone cysts – a prospective analysis of 19 lesions. Virchows Arch 2021; 479: 795–802. 10.1007/s00428-021-03130-5 34089379

[10] Cordier F , Creytens D . Unravelling the USP6 gene: an update. J Clin Pathol 2023; 76: 573–577. 10.1136/jcp-2023-208896 37328256

[11] Wang J‐C , Li W‐S , Kao Y‐C , *et al*. Clinicopathological and molecular characterisation of USP6‐rearranged soft tissue neoplasms: the evidence of genetic relatedness indicates an expanding family with variable bone‐forming capacity. Histopathology 2021; 78: 676–689. 10.1111/his.14268 33000481

[12] Cordier F , Van Dorpe J , Sciot R , *et al*. A morphomolecular study of 175 ‘USP6‐associated neoplasms’: the USP6 fusion partner strongly depends on morphology and location. Histopathology 2025; 88: 898–910. 10.1111/his.70057 41293881

[13] Sun L , Zhao Z , Zhu Y . USP6‐associated neoplasm as a tentative subset of postoperative spindle cell nodule. Histopathology 2023; 82: 587–595. 10.1111/his.14841 36404122

[14] Saoud C , Agaimy A , Stoehr R , *et al*. Nodular fasciitis: a case series unveiling novel and rare gene fusions, including two cases with aggressive clinical behavior. Virchows Arch 2025; 486: 1235–1245. 10.1007/s00428-025-04040-6 PMC12704652 39912885

[15] Kashofer K , Gornicec M , Lind K , *et al*. Detection of prognostically relevant mutations and translocations in myeloid sarcoma by next generation sequencing. Leuk Lymphoma 2018; 59: 501–504. 10.1080/10428194.2017.1339879 PMC5743000 28633614

[16] Hench J , Hultschig C , Brugger J , *et al*. EpiDiP/NanoDiP: a versatile unsupervised machine learning edge computing platform for epigenomic tumour diagnostics. Acta Neuropathol Commun 2024; 12: 51. 10.1186/s40478-024-01759-2 PMC10993614 38576030

[17] Dworak DP , Maltry AC , Bell WR . Nodular fasciitis of the periorbital soft tissue in an adolescent confirmed by USP6 gene rearrangement. Am J Ophthalmol Case Rep 2022; 26: 101496. 10.1016/j.ajoc.2022.101496 PMC8971580 35372714

[18] Parkinson B , Patton A , Rogers A , *et al*. Intraneural nodular fasciitis of the femoral nerve with a unique CTNNB1::USP6 gene fusion: apropos of a case and review of literature. Int J Surg Pathol 2022; 30: 673–681. 10.1177/10668969221080064 35175167

[19] Stražar K , Šekoranja D , Matjašič A , *et al*. Intraarticular nodular fasciitis – detection of USP6 gene fusions in three cases by targeted RNA sequencing. Virchows Arch 2021; 478: 1117–1124. 10.1007/s00428-020-02991-6 33404853

[20] Vuong M , Mejbel HA , Mackinnon AC , *et al*. Nodular fasciitis of the buccal mucosa with a novel USP6 gene rearrangement: a case report and review of the literature. Head Neck Pathol 2024; 18: 79. 10.1007/s12105-024-01687-6 PMC11339223 39167308

[21] Sawamura C , Gokita T , Ishikawa A , *et al*. An aggressive nodular fasciitis lesion protruding from the palm (USP6 gene fusion helps differentiate from sarcomas): a case report. Mol Clin Oncol 2021; 14: 10. 10.3892/mco.2020.2172 PMC7709569 33282285

[22] Ovechko V , Peterson JM , Hasan H , *et al*. Recurrent nodular fasciitis of the oral mucosa: case report and review of the literature. Int J Surg Pathol 2025; 33: 992–1000. 10.1177/10668969241297258 39552462

[23] Guo R , Wang X , Chou MM , *et al*. PPP6R3‐USP6 amplification: novel oncogenic mechanism in malignant nodular fasciitis. Genes Chromosomes Cancer 2016; 55: 640–649. 10.1002/gcc.22366 27113271

[24] Teramura Y , Yamazaki Y , Tanaka M , *et al*. Case of mesenchymal tumor with the PPP6R3‐USP6 fusion, possible nodular fasciitis with malignant transformation. Pathol Int 2019; 69: 706–709. 10.1111/pin.12851 31538390

[25] Malik F , Wang L , Yu Z , *et al*. Benign infiltrative myofibroblastic neoplasms of childhood with USP6 gene rearrangement. Histopathology 2020; 77: 760–768. 10.1111/his.14182 32583473

[26] Wang C , Wang W , Xu R , *et al*. Case report: large‐size intramuscular nodular fasciitis, a challenging histopathologic diagnosis confirmed by molecular detection of USP6 gene rearrangement: case report and literature review. Pathol Oncol Res 2023; 29: 1610785. 10.3389/pore.2023.1610785 PMC9894875 36741963

[27] Arcovito G , Crucitta S , Del Re M , *et al*. Recurrent USP6 rearrangement in a subset of atypical myofibroblastic tumours of the soft tissues: low‐grade myofibroblastic sarcoma or atypical/malignant nodular fasciitis? Histopathology 2024; 85: 244–253. 10.1111/his.15196 38651320

[28] Tomassen T , van de Ven C , Anninga J , *et al*. Nodular fasciitis with malignant morphology and a COL6A2–USP6 fusion: a case report (of a 10‐year‐old boy). Int J Surg Pathol 2021; 29: 642–647. 10.1177/1066896921996045 PMC8343208 33625261

[29] Qiu Y , Peng R , Chen H , *et al*. Atypical nodular fasciitis with a novel PAFAH1B1‐USP6 fusion in a 22‐month‐old boy. Virchows Arch 2021; 479: 623–629. 10.1007/s00428-020-02961-y 33161462

[30] Papke DJ , Oliveira AM , Chou MM , *et al*. Morphologically malignant nodular fasciitis with CALD1‐USP6 fusion. Virchows Arch 2021; 479: 1007–1012. 10.1007/s00428-021-03149-8 34213575

[31] Salah M , Rimani M , Benjelloun L , *et al*. Pediatric nodular fasciitis of the orofacial region: a rare case report and literature review. Int J Surg Case Rep 2025; 135: 111929. 10.1016/j.ijscr.2025.111929 PMC12464542 40967117

[32] Lenz J , Michal M , Svajdler M , *et al*. Novel EIF5A‐USP6 gene fusion in nodular fasciitis associated with unusual pathologic features: a report of a case and review of the literature. Am J Dermatopathol 2020; 42: 539–543. 10.1097/DAD.0000000000001602 31880592

[33] Cloutier JM , Kunder CA , Charville GW , *et al*. Nodular fasciitis of the breast: clinicopathologic and molecular characterization with identification of novel *USP6* fusion partners. Mod Pathol 2021; 34: 1865–1875. 10.1038/s41379-021-00844-4 34099872

