# Supplementary material for: The molecular landscape of nodular fasciitis: novel USP6 fusions, genomic stability, and an exploratory clinicopathological model – a 20‐year retrospective study
Source: J Pathol Clin Res. 2026 Oct 9;12(6):e70117. doi: 10.1002/2056-4538.70117 (PMC13650599; doi:10.1002/2056-4538.70117)
Supplement: Supplementary file 1 [file CJP2-12-e70117-s001.pdf]

The molecular landscape of nodular fasciitis: novel USP6 fusions, genomic stability, and an exploratory clinicopathological model – a 20-year retrospective study

K Skok *et al. J Pathol Clin Res.* https://doi.org/10.1002/2056-4538.70017

Supplementary Figure S1
Supplementary Tables S1 and S2

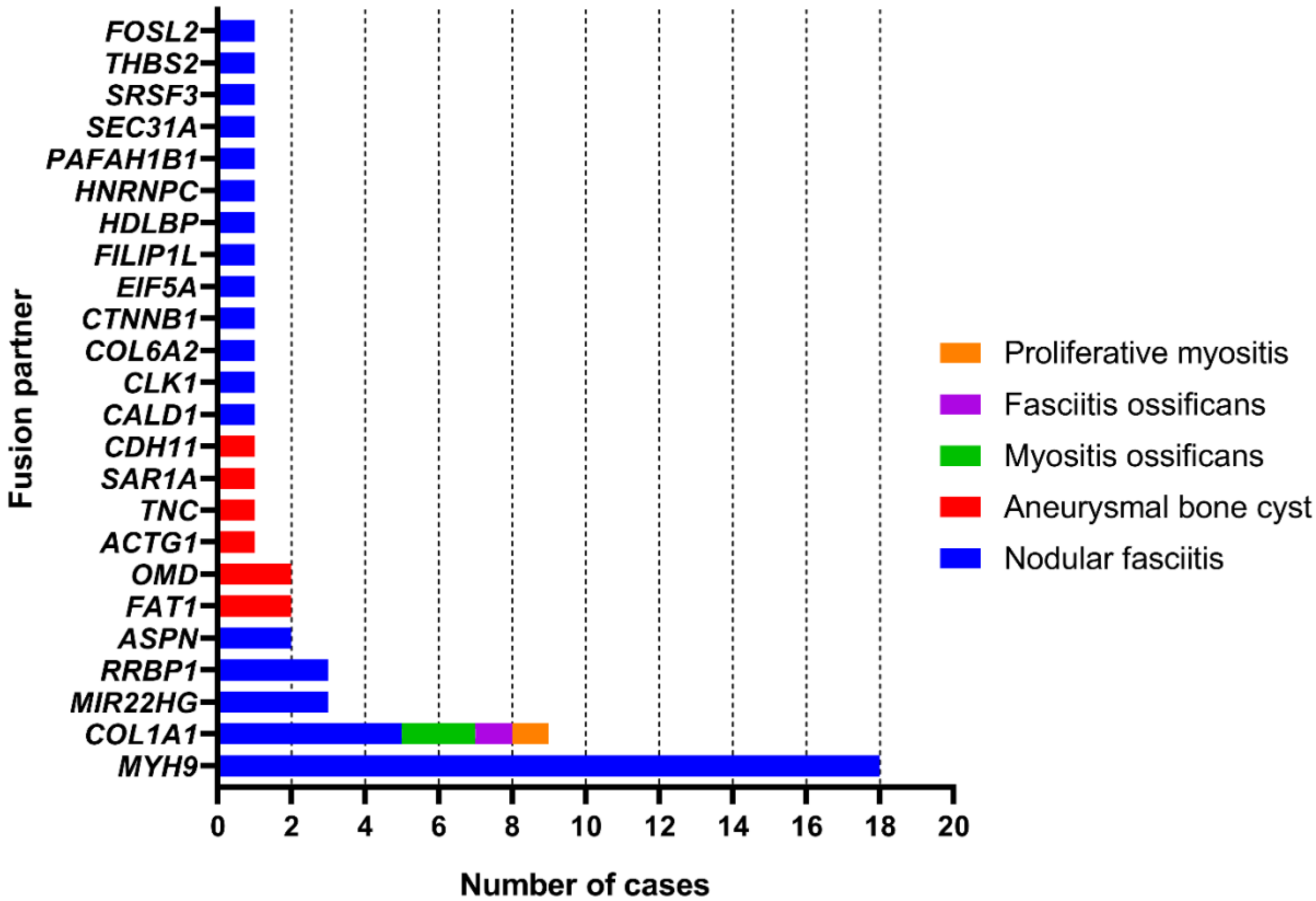

Figure S1. Distribution of *USP6* fusion partners across benign fibro-osseous and fibro-myofibroblastic lesions. Horizontal bar chart showing the number of reported *USP6*-rearranged cases categorized by fusion partner gene (y-axis). Five diagnostic entities are represented and color-coded: nodular fasciitis (blue), aneurysmal bone cyst (red), myositis ossificans (green), fasciitis ossificans (orange), and proliferative myositis (purple). Among all lesions, *MYH9* and *COL1A1* are the most frequent fusion partners, predominantly associated with NF and less commonly with other *USP6*-associated entities. Labels on the x-axis indicate the number of cases per fusion partner.

Table S1. Distribution of nodular fasciitis (NF) cases included in the study and the molecularly investigated subset according to referral source.

Cohort	N of cases	Consultation referrals	Cases from the university hospital	Cooperation partner (KAGes)
Overall NF cohort	283	137 (48.4%)	91 (32.2 %)	55 (19.4 %)
Molecularly investigated NF subset	44	31 (70.5 %)	7 (15.9 %)	6 (13.6 %)

Table S2. Clinical characteristics of the remaining *USP6*-associated lesions found during database review. ABC = aneurysmal bone cyst; MO = myositis ossificans; FO = fasciitis ossificans; PM = proliferative myositis; M = male; F = female. *Lesion size represents maximum dimension; only cases with documented measurements were included.

**Variable**	**Aneurysmal bone cyst (ABC, *n* = 8)**	**Myositis ossificans (MO, *n* = 2)**	**Fasciitis ossificans (FO, *n* = 1)**	**Proliferative myositis (PM, *n* = 1)**
Age, years	Median 17.5 (range 9–23)	22 and 40	22	21
Sex	4 M / 4 F	2 F	1 M	1 F
Lesion size*	Median ~1.4 cm (range ~1–1.5 cm)	4 cm and 3,5 cm	5 cm	7 cm
Anatomic region	Upper ext.: 2 (25.0%)<br>Lower ext.: 3 (37.5%)<br>Trunk: 3 (37.5%)	Trunk: 2 (100%)	Thigh	Axilla
Behavior	Osseous lesions, all treated locally	Bone-forming variants	Localized lesion	Localized, no aggressive features
